# Revisiting CN^−^ Formation Mechanisms in Electron Collisions with Benzonitrile

**DOI:** 10.1002/cphc.202500206

**Published:** 2025-09-28

**Authors:** Rodrigo Rodrigues, Mónica Mendes, Daniel Bou‐Debes, João Ameixa, Ali Kamali, Oddur Ingólfsson, Samuel Eden, Lucas M. Cornetta, Filipe Ferreira da Silva

**Affiliations:** ^1^ CEFITEC Departamento de Física NOVA School of Science and Technology Universidade NOVA de Lisboa Campus de Caparica 2829‐516 Caparica Portugal; ^2^ School of Physical Sciences The Open University Walton Hall Milton Keynes MK7 6AA UK; ^3^ School of Life, Chemical, and Health Sciences The Open University Walton Hall Milton Keynes MK7 6AA UK; ^4^ Science Institute and Department of Chemistry University of Iceland Dunhagi 3 Reykjavik IS‐107 Iceland; ^5^ Instituto de Física, Universidade de São Paulo Rua do Matão 1731 São Paulo 05508‐090 Brasil

**Keywords:** benzonitrile, cyano anions, dissociative electron attachment, Schwinger multichannel methods

## Abstract

Radiation‐induced processes in the aromatic cyano compound benzonitrile have attracted renewed interest since its detection in the interstellar medium in 2018, and recent studies have elucidated dissociative ionization pathways leading to species such as CN^•^ and HCN, which can play important roles in interstellar chemistry. This work explores negative ion formation from benzonitrile upon electron attachment with mass spectrometry experiments and the most extensive theoretical study to date of the underlying negative ion states and their respective dissociative relaxation pathways. The measurements confirm the previously reported CN^−^ formation at a collision energy of 3.0 eV as well as formation of the dehydrogenated parent anion and phenyl anion and CN^−^ formation in the 7–10 eV energy range. Threshold energies for these dissociation channels are reported at the G4(MP2) level of theory for the first time. Furthermore, by using both scattering calculations and bound state techniques, CN^−^ formation at around 3.0 eV may proceed from a ^2^B_1_, π_4_* shape resonance through nonadiabatic coupling with the σ*, C—CN state. In the 7–10 eV range, complete active space plus second‐order perturbation (CASPT2) calculations suggest strong contributions from core excited π_4_* and σ* resonances.

## Graphical abstract

This study explores negative ion formation via electron attachment, revealing CN^−^ formation at 3.0 and 7–10 eV. Dissociation pathways are analyzed using G4(MP2) and CASPT2 calculations, highlighting the coupling between the π4* and σ* resonances critical for interstellar chemistry.

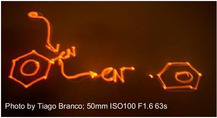

## 1 Introduction

Benzonitrile, also known as phenyl cyanide, is an organic nitrile with the molecular formula C_6_H_5_CN. It has various industrial applications, including its use as a chemical intermediate in the synthesis of pharmaceuticals,^[^ ^1^ ^]^ agrochemicals,^[^ ^2^ ^]^ and dyes.^[^ ^3^ ^]^ Furthermore, it has caught the attention of astrochemists and astrophysicists due to its identification, by McGuire and colleagues in 2018, in the cold core of the Taurus Molecular Cloud.^[^ ^4^ ^]^ This is an important discovery, as the presence of benzonitrile, together with other complex molecules such as polycyclic aromatic hydrocarbons (PAHs), indicates rich and dynamic interstellar chemistry—a chemistry driven by UV radiation, cosmic rays, electrons, and other secondary products of radiation‐induced processes.^[^ ^5^, ^6^ ^]^ Recently, other polycyclic aromatic cyano compounds have been identified in the Taurus Molecular Cloud 1 (TMC‐1), including all configurational isomers (1‐, 2‐, and 4‐) of cyanopyrene,^[^ ^7^, ^8^ ^]^ as well as the CN‐substituted seven‐ring cyanocoronene (C_2__4_H_1__1_CN).^[^ ^9^ ^]^ Interestingly, the estimated abundance ratio of the configurational isomers of cyanopyrene in TMC‐1 (1:2:1) matches that of the substitution sites of pyrene, suggesting a kinetically controlled CN substitution in the low‐temperature environment of TMC‐1. In fact, pyrene (C_1__6_H_1__0_) has been estimated to account for up to 0.1% of the total carbon budget of TMC‐1, and anionic PAHs are discussed as the main negative charge carriers in TMC‐1.^[^ ^9^, ^10^ ^]^

Elucidating the chemical pathways leading to complex organic molecules in the interstellar medium (ISM) is an important part of the effort to understand the molecular origins of life, and nitrogen‐containing species must play a role in these processes. CN^−^ is particularly interesting due to its unexpectedly high abundance in the ISM,^[^ ^11^ ^]^ and aromatic cyano compounds including benzonitrile are sources for interstellar CN^−^ formation. This may thus play a significant role in the dynamic production and decay of complex nitrogen‐containing molecules in some interstellar environments. In addition, experiments aimed at recreating discharge‐driven chemistry in conditions approximating Titan's upper atmosphere have identified benzonitrile production.^[^ ^12^ ^]^ Characterizing electron interactions with benzonitrile may therefore have applications for modelling the production of reactive species in the atmospheres of planetary bodies, as well as the possible formation pathways of more complex stable molecules.

Various electron ionization and photoionization studies have been carried out on benzonitrile in the last few years with astrophysical/astrochemical motivations, characterizing the production of positively charged and neutral fragments.^[^ ^6^, ^13^, ^14^, ^15^ ^]^ Conversely, three experimental studies on negative ion formation from benzonitrile are available in the literature: an electron transmission (ET) study conducted by Burrow et al.^[^ ^16^ ^]^ in 1992 on a series of nitriles and a dissociative electron attachment (DEA) study conducted by Heni and Illenberger in 1986 also reported on several other CN‐containing compounds.^[^ ^17^ ^]^ Recently, Abdoul‐Carime et al.^[^ ^18^ ^]^ have described negative ion formation of electron interactions with benzonitrile and benzonitrile mixed with calibration gas CCl_4_, reporting the threshold enthalpies for CN^−^, C_6_H_5_^−^, and C_6_H_4_CN^−^ formation calculated at *ω*B97*x*/aug‐cc‐pvtz level of theory. Electron attachment to pentafluoro benzonitrile has also been studied by Langer and co‐authors.^[^ ^19^ ^]^ However, unlike benzonitrile, electron attachment to pentafluoro benzonitrile supports stable parent anion due to its higher electron affinity (1.11 ± 0.11 eV^[^ ^20^ ^]^ in contrast to that of benzonitrile, reported as 0.26 ± 0.02 eV^[^ ^21^ ^]^ and 0.239 ± 0.005 eV).^[^ ^22^, ^23^ ^]^ As with the previous experimental work, there are few papers in the literature that address negative ion formation from benzonitrile from the theoretical standpoint. Burrow et al.^[^ ^16^ ^]^ applied molecular orbital theory to assign their ET spectral features to π* valence anionic The four observed structures were assigned to shape resonances at 0.57 eV (^2^A_2_), 2.57 eV (^2^B_2_), 3.19 eV (^2^B_1_), and 4.6 eV (^2^B_1_). Furthermore, they described the lowest‐lying state—a ^2^ *B*_1_ state—as a shallow bound state, since it was not observed in the ET measurements. Kirnosov et al.^[^ ^23^ ^]^ calculated a dipole‐bound state (DBS), bounded by 19 meV. More recently, Gulania et al.^[^ ^24^ ^]^ reported calculations on the energies of both the DBS and the lowest‐lying valence anion state ^2^B_1_, also referred to as π_1_*. Although their findings show the vertical attachment energy (VAE) of −0.024 eV for the DBS, they observed that the π_1_* is electronically unbound, which introduces an additional complexity to the landscape of benzonitrile anions. It is relevant to point out that even though the π_1_* state presents a positive VAE, the anion is adiabatically bound due to differences in the zero‐point energy corrections that slightly surpass the electronic contribution.

In the present contribution, we report DEA experiments that verify the only previous measurements of this kind by Heni and Illenberger^[^ ^17^ ^]^ and Abdoul‐Carime et al.^[^ ^18^ ^]^ The experimental energies for CN^−^ formation compared with threshold energies calculated at the G4(MP2) level of theory, and we report the first quantum scattering calculations to characterize the shape resonances for low‐energy electron attachment to benzonitrile. This represents a significant step forward for the overall theoretical description of the molecule's low‐lying valence anion states, and second‐order perturbation (CASPT2) calculations^[^ ^25^, ^26^ ^]^ were used to further explore contributions from higher energy electronically excited states.

## 2 Experimental Section

### 2.1 Experimental Methods

DEA experiments were carried out using a crossed electron‐molecule beam system at the CEFITEC, Nova University Lisbon. The setup has been described in detail elsewhere.^[^ ^27^ ^]^ Briefly, it consisted of a trochoidal electron monochromator (TEM),^[^ ^28^ ^]^ a reflectron time‐of‐flight (ToF) mass spectrometer (MS) in orthogonal geometry, and an effusive target sample inlet. Electrons emitted by a harpin tungsten filament passed through the TEM to provide a quasi monoenergetic electron beam. The effusive beam of the target benzonitrile molecules crossed the electron beam orthogonally. The ions formed in the resultant electron‐molecule collisions were then extracted by a weak field (≈1 V cm^−1^) into the reflectron ToF‐MS and detected with a multichannel plate detector. The electron beam current was measured at a Faraday plate and was typically 100 nA for an electron energy of ≈0 eV. The electron energy scale was calibrated using the well‐known, resonant Cl^−^ DEA signal from CCl_4_ at 0 and 0.75 eV.^[^ ^29^ ^]^ The CCl_4_ calibration scan was obtained before and after the measurement to verify the stability of the electron beam during the measurement. The electron energy resolution was obtained as 250 meV from the FWHM of the ≈0 eV Cl^−^/CCl_4_ resonance. The background pressure in the system was 10^−6^ Pa, after removing CCl_4_ calibration gas, leading to a negligible background signal; the pressure during the benzonitrile measurements was 5 × 10^−4 ^Pa. The benzonitrile anhydrous ≥99% was purchased from Sigma–Aldrich. The sample was purified in four freeze–pump–thaw cycles. The benzonitrile sample was heated up to 120 °C to get enough molecular density for the measurements.

### 2.2 Theoretical Methods

Quantum chemical calculations have been carried out with the objective of identifying the electron attachment process and the dissociative dynamics of transient anions in benzonitrile. These were based on both scattering calculations and bound state techniques. First, calculating the integral cross section for the collision between low‐energy electrons and benzonitrile provided the characterization of low‐lying anionic states. Particularly for π* anionic states, an additional calculation was performed based on scaled Hartree–Fock (HF) virtual orbital energies (VOEs), and they were used as a comparison.^[^ ^23^ ^]^ This approach estimated the VAE by scaling the HF eigenvalues of the unoccupied π* orbitals of the neutral reference according to the relation VAE (eV) = 0.6479 × VOE (eV) − 1.4298. Second, among other important information, such as reaction rate or electron affinity of the fragments, reaction energy thresholds provided the energetic viability between the anionic states and different dissociation channels. The specific methods are described below.

For the scattering calculations, we have employed the Schwinger multichannel (SMC) method implemented with the Bachelet–Hamann–Schlüter^[^ ^24^, ^25^ ^]^ pseudopotentials (SMCPP). The scattering calculations were conducted within the fixed‐nuclei approach, and since the equilibrium geometry of neutral benzonitrile belonged to the C_2v_ point group, symmetry operations have been explored throughout the entire study. For instance, the cross sections have been decomposed into A_1_, A_2_, B_1_, and B_2_ symmetry components. For the heavy atoms (all but hydrogen), the basis sets used for the valence electrons were an uncontracted 5*s*5*p*2*d* set for the carbon atoms and an uncontracted 6*s*5*p*2*d* set for the nitrogen as generated by Bettega et al.^[^ ^30^ ^]^ The exponents of the basis sets for the carbon and nitrogen atoms are summarized in **Table** **1**. For the hydrogen, the 3*s*1*p* Dunning basis set^[^ ^31^ ^]^ was employed. Further details of the SMCPP variational approach and its implementation can be found elsewhere.^[^ ^32^ ^]^

**Table 1:** Exponents of the basis functions employed for the carbon and nitrogen atoms.

Function type	C	N
*s*	12.496280	17.569870
	2.470286	3.423613
	0.614028	0.884301
	0.184028	0.259045
	0.039982	0.053066
	–	0.013266
	5.228869	7.050692
	1.592058	1.910543
	0.568612	0.579261
	0.210326	0.165395
	0.072250	0.037192
*d*	0.603592	0.403039
	0.156753	0.091192

Although SMCPP allowed the calculation for the elastic and inelastic channels, we focused on the description of the lower‐lying anion states. Therefore, only the elastic channel has been considered. The method was based on the expansion of the scattering wave function in the configuration state function (CSF) space. The CSFs were functions of N + 1 electrons, whose strategic choice in turn defined the so‐called static exchange (SE) and SE plus polarization (SEP) approximations. The former only considered CSFs given by

(1) $$\left|\psi_{i}>=A\left[\left|\varphi_{0}>⊗\right|\varphi_{i}>\right]\right|$$

where *A* is the antisymmetrization operator, | *Φ*_0_ *>* is the target ground state obtained in the HF approximation, and | *φ*_i_ *>* denoted the scattering orbital, labeled by the index i. Consequently, the SE scheme neglected correlation–polarization effects. In contrast, in the SEP expansion, the CSFs were doubled‐indexed functions of the kind

(2) $$\left|\psi_{ij}>=A\left[\left|\varphi_{i}>⊗\right|\varphi_{j}>\right]\right|$$

where | *Φ*_i_ *>* is a single excited target state with either singlet or triplet spin coupling. In other words, each CSF in the SEP approximation was characterized by three one‐electron functions, namely, particle, hole, and scattering orbital. Instead of virtual orbitals from the HF calculation, modified virtual orbitals (MVO)^[^ ^33^ ^]^ were generated from the cationic Fock operators with charge +4 and used for both particle and scattering orbitals. Setting up the CSF space was crucial for the SEP approximation to provide a balanced description of the anion states in relation to the neutral species, and the energy criteria proposed by Kossoski et al.^[^ ^34^ ^]^ have been applied for that. The strategy was based on the orbital's eigenvalues and a user‐defined cutoff energy *Δ*, in such a way that only configurations satisfying *ε*_*p*_ *‐ ε*_*h*_ *+ ε*_s_ *< *Δ were included in the CSF space. The chosen values for *Δ* were such that the numbers of CSFs were 15 449, 14 392, 14 692, and 15 162 for A_1_, A_2_, B_1_, and B_2_ components, respectively.

The equilibrium geometry and vibrational analysis of neutral benzonitrile were obtained with density functional theory (DFT), employing the hybrid functional B3LYP together with the aug‐cc‐pVTZ basis set, as implemented in the *gaussian*16 package.^[^ ^35^ ^]^ The dissociation threshold for a particular reaction was defined as the free energy difference between products and the reactant. These quantities, for the relevant dissociation channels, were obtained within G4(MP2) composite method,^[^ ^36^, ^37^ ^]^ as implemented in *gaussian*16. Finally, the CASPT2 calculations for the anion species were addressed using the ANO‐S basis set and considering an active space comprising 9 electrons in 15 orbitals, and all core orbitals were kept frozen. The CASPT2 calculations were performed with the OpenMolcas package.^[^ ^38^ ^]^

## 3 Results and Discussion

**Figure 1:**
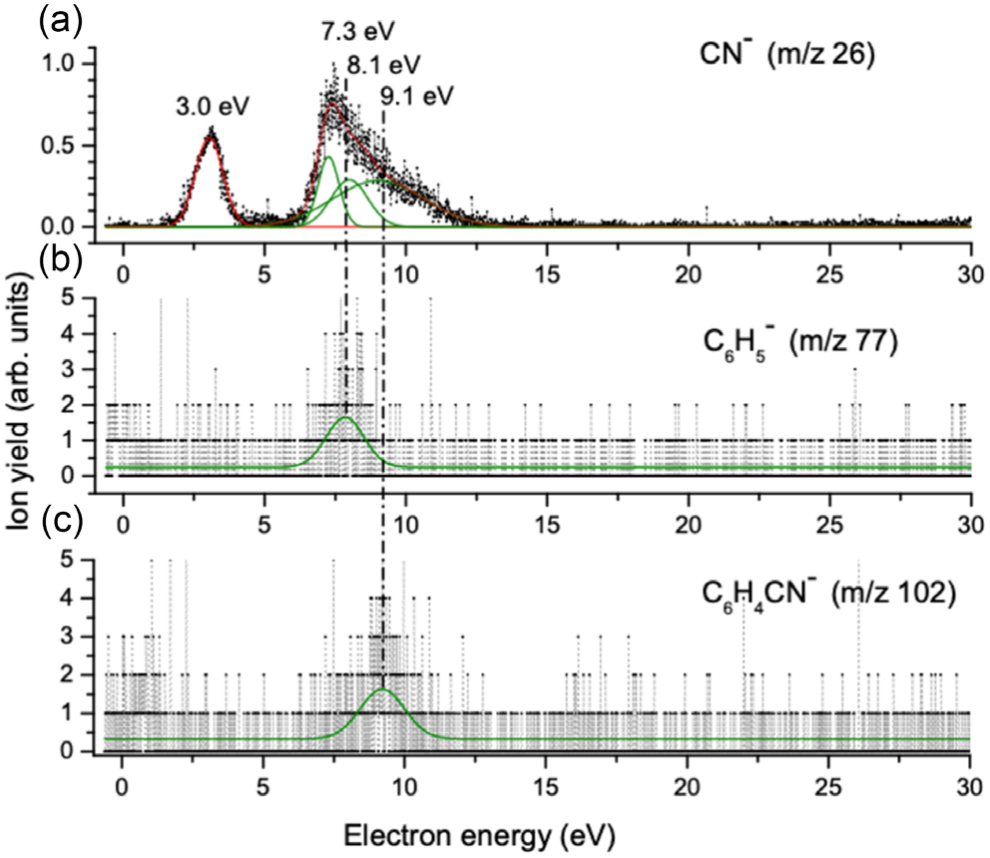
Energy dependence for the three observed fragments formed in DEA to benzonitrile in the energy range from 0 to 30 eV: a) formation of CN^−^, b) formation of the phenyl anion, and c) formation of the dehydrogenated parent anion. The individual contributions to the ion yields are reproduced with Gaussian fits, as is discussed in the text. Green curves show the individual contributions, with their maxima noted in the top panel. The red curve in panel (a) shows the sum of the individual components. The weak signal between 0 and 1 eV electron energy is an artefact due to multiple reflection of very low energy electrons in the lenses of the electron gun.

**Figure** **1**a shows the energy dependence of the ion yield for the formation of CN^−^ upon electron attachment in the incident electron energy range from about 0 to 30 eV. This extended energy range is chosen to also explore potential anion formation through dipolar dissociation (DD). The energy dependence of the ion yields for the two less intense fragments, the phenyl anion and the dehydrogenated parent anion, is shown in Figure 1b,c, respectively. To better determine the onset, maxima, and composition of the respective ion yields, these are fitted using Gaussian functions as discussed below. In the explored energy range, CN^−^ is observed through a resonant contribution centered at 3.0 ± 0.3 eV and a broader composite contribution peaking at 7.3 ± 0.3 eV but extending to above 12 eV electron incident energy. The other two negative ion fragments observed, the dehydrogenated parent anion and the phenyl anion, are observed with significantly lower intensity and have resonant structures peaking at 9.1 ± 0.3 and 8.1 ± 0.3 eV, respectively. A good fit to the broad, high‐energy CN^−^ contribution is achieved by incorporating resonant contributions from both these channels as well as the resonant contribution centered at 7.3 eV. Table 1 compares the threshold energies for the individual processes calculated at the G4(MP2) level of theory, with the peak maxima for the individual contributions to the respective ionic fragments as determined by the Gaussian fits to the ion yield curves. The values are compared to the reaction enthalpies reported by Abdoul‐Carime et al.^[^ ^18^ ^]^ and the experimental peak values reported by Heni and Illenberger.^[^ ^17^ ^]^ Also shown are the calculated thresholds for the parent ion formation (the negative value of the adiabatic electron affinity) and for CN^−^ formation through DD, though no signs of this process are apparent in any of the three ion yields observed from benzonitrile.

Qualitatively, the current measurements agree with those published by Heni and Illenberger,^[^ ^17^ ^]^ and though the relative intensities of the individual fragments differ, the maxima of the Gaussian fits to the respective resonant contributions agree within the reported energy resolutions.

It is clear that DEA to benzonitrile occurs in two energy regimes. The first is centered at 3.0 eV, as determined by the maximum of the Gaussian fit to the first contribution in the CN^−^ ion yield. For this channel, the appearance energy (AE; the onset of the ion yield) was found to be 1.6 ± 0.3 eV, which agrees within the confidence limits with the free energy threshold of 1.84 eV, calculated at the G4(MP2) level of theory for this process. As expected, the enthalpy values calculated by Abdoul‐Carime et al.^[^ ^18^ ^]^ of 2.06 eV is slightly higher. As may be seen in Table 1, this is the case for all common threshold values calculated. Using the difference between the literature values for the C—CN bond dissociation energy reported as 5.759^[^ ^39^ ^]^ and 5.737 eV,^[^ ^40^ ^]^ and the well‐established 3.862 ± 0.004 eV^[^ ^41^ ^]^ adiabatic electron affinity of the CN neutral, gives a threshold energy of 1.9 eV. This is in agreement with our G4(MP2) value and within the confidence limits for the experimentally obtained AE. Apart from this channel centered at 3.0 eV, CN^−^ is also formed via a broad, composite contribution peaking at about 7.3 eV and extending to above 12 eV. In this energy range, though with low intensity, resonant contribution in the DEA phenol anion yield is observed to peak at about 8.1 eV and in the ion yield of the dehydrogenated benzonitrile anion at 9.1 eV. We anticipate that the underlying transient negative ion leading to these contributions primarily decay to form the CN^−^ anion.

While the 8.1 ± 0.3 eV contribution to the CN^−^ yield can be attributed to single‐bond rupture with dominating charge retention on the CN fragment and to a lesser extent on the complementary phenol moiety, the 9.1 ± 0.3 eV contribution is associated not only with CN^−^ formation but also leads to the dehydrogenated parent anion formation. In addition, CN^−^ is formed in this energy range through a lower‐lying resonance. This contribution peaks at about 7.3 ± 0.3 eV in the CN^−^ ion yield as demonstrated by the Gaussian fit of these three components to the CN^−^ contribution above about 6 eV.

**Table** **2** compares the first valence anion states of benzonitrile obtained with the SMCPP method and the values obtained by the scaled VOE approach, along with the respective resonance energies observed in ET experiments by Burrow et al.^[^ ^16^ ^]^ Also shown is the experimental peak position of CN^−^ formation in this energy range as determined by the Gaussian fit.

**Table 2:** G4(MP2) values for the reaction free energy thresholds for the fragmentation channels observed in DEA to benzonitrile, for formation of the parent anion and DD leading to CN^−^ formation, and the reaction enthalpies reported in ref. [18]. Also shown are the peak positions as determined by the Gaussian fits to the respective ion yield curves in the present DEA experiments and the estimated peak positions from ref. [17]. All values are given in eV.

Products	$\Delta G_{th}$ [eV]G4[MP2]	$\Delta G_{th}$ [eV] Ref.[18]	Peak positions [±0.3 eV]	Peak positions Ref.[17]
M ^−^	−0.26	–	Not observed	Not observed
[M − CN] ^−^ + CN	4.57	5.21	8.1	7.0–8.0
[M − CN] + CN ^−^	1.84	2.06	3.0, 7.3, 8.1, 9.1	3.0, 7.0–8.0, 9.0–10.0
[M − H] ^−^ + H	2.95	3.18	9.1	7.0–8.0, 8.0–9.0
[M − CN]^+^ + CN^−^	10.12	–	Not observed	Not observed

**Figure 2:**
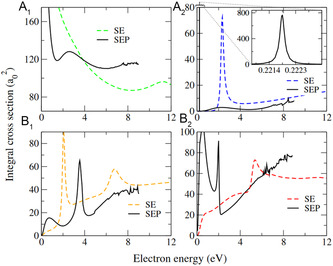
SMCPP integral cross section for low‐energy electron scattering by benzonitrile, separated by the symmetry components of the irreducible representation of the C_2v_ point group. The colored dotted lines denote the calculation performed at the SE level of approximation, while the solid black lines denote these are computed at the SEP level of approximation. All cross sections are reported in units of a_0_^2^.

**Figure 3:**
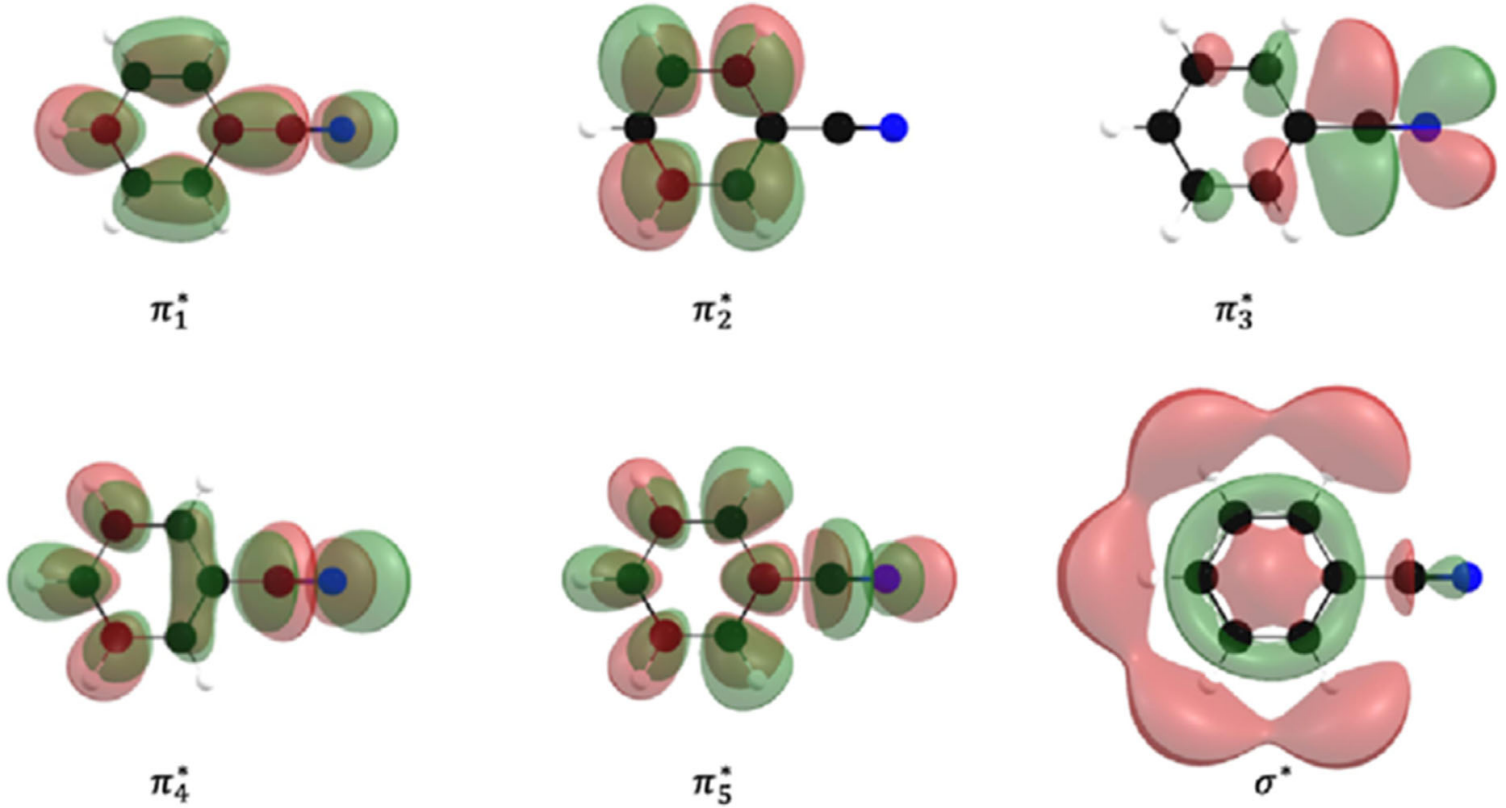
Contour plots of the first six LUMOs of benzonitrile. The symmetries of the resonances associated with single‐electron occupation of these and the respective VAEs are summarized in Table 2.

The calculations of the integral cross section for electron scattering, shown in **Figure** **2**, allow description of some of the low‐lying anionic states of benzonitrile. For reference, the isosurfaces of the respective lowest unoccupied molecular orbital (LUMOs) of the neutral associated with the formation of these resonances are shown in **Figure** **3**.

The first valence anionic state of benzonitrile is found to be a shallow, bound π_1_* ^2^B_1_ state. For this anionic state, single‐point calculations at the DFT/B3LYP/aug‐cc‐pVTZ level of theory give an estimate of the VAE of ≈0.04 eV, relative to the neutral ground state energy, and the VOE scaling approach gives a VAE of ≈0.01 eV. Consistently, the G4(MP2) calculations give a value of about 0.26 eV for the respective adiabatic electron affinity, which agrees with the experimental value of 0.26 eV reported by Zlatkis et al.^[^ ^21^ ^]^ as well as that of 0.258 ± 0.018 eV determined by Wentworth et al.^[^ ^22^ ^]^

In good agreement with the current results, Burrow et al.^[^ ^16^ ^]^ anticipate this ^2^B_1_, lowest‐lying anionic resonance of benzonitrile, to be bound by only few tens of eV and thus not observed in the respective ET spectra.

The current SMCPP‐SEP approach, in contrast, gives a VAE of −0.46 eV for this π_1_* state. Such underestimation of the VAE based on the scattering calculations is, however, expected, since the number of CSFs in the SEP approach was set to reasonably describe the resonances near the first DEA peak of the CN^−^ channel, i.e., from 3 to 4 eV. Furthermore, the resonant structure associated with this π_1_* ^2^B_1_ state is observed in the SE cross section around 2.1 eV (orange dashed line in the bottom left panel of Figure 2), which indicates a stabilization of more than 2.5 eV in going from the SE to the SEP approach.

The other signatures presented in Figure 2 relate to four π* and one σ* shape resonances. In the SMCPP‐SEP calculations, only three of the four π* states have been clearly identified, namely, at 0.22 eV (π_2_*, A_2_), 1.99 eV (π_3_*, B_2_), and 3.76 eV (π_4_*, B_1_), while the VOE scaling approach places these states at 0.63, 2.13, and 3.07 eV, respectively (see **Table** **3**). This correlates well with the ET spectra from Burrow et al.,^[^ ^16^ ^]^ which place the first ^2^A_2_ (π_2_*) negative ion resonance at 0.54 eV, the π_3_ * ^2^B_2_ resonance at 2.57 eV and the second ^2^B_1_ resonance (π_4_*) at 3.19 eV.

**Table 3:** Energies of the low‐lying valence anion states of benzonitrile, obtained with the SMCPP method compared with values obtained by the VOEs and those determined by ETS by Burrow et al.^[^ ^16^ ^]^ All values are relative to the neutral ground state of benzonitrile and are given in eV. Also shown is the present lowest energy experimental DEA resonance peak position for CN^−^ production.

State	Sym.	SMCPP	VOE scaling	Present DEA exp.	ETS^[^ ^16^ ^]^
π_1_*	B_1_	−0.49	0.01	–	–
π_2_*	A_2_	0.22	0.63	–	0.57
π_3_*	B_2_	1.99	2.13	–	2.57
π_4_*	B_1_	3.76	3.07	3.0	3.19
π_5_*	B_1_	–	5.01	–	4.62
σ*	A_1_	2.83	–	–	–

The remaining π_5_* ^2^B_1_ state is expected to be found as a broader structure around 6 eV, as supported by further analysis of the electronic Hamiltonian, although the numerically spurious behavior of the cross section in that energy range makes it difficult to have a clear characterization of this particular resonance. The alternative procedure based on VOE scaling provides a relatively good estimation of π* shape resonances of small unsaturated compounds and places the π_5_* ^2^B_1_ resonance at 5.01 eV, which is in fairly good agreement with the ET experiments^[^ ^16^ ^]^ which place this ^2^B_1_ resonance at 4.62 eV.

Lastly, a broad σ* shape resonance (A_1_) is found at 2.83 eV (Table 2), which presents a dissociative character along the C—CN bond. This component is not identified in the electron transmission spectroscopy (ETS) from Burrow et al.,^[^ ^16^ ^]^ which may be due to the short lifetime of this σ* shape resonance and/or its energetic proximity to the π_3_* and π_4_* resonances masking its contribution in the ETS.

It is clear from Table 2 that the lowest energy DEA channel for benzonitrile is that of the CN^−^ formation, with a threshold energy of 1.84 eV. This is well above the VAE leading to the formation of the π_1_* ^2^B_1_ and π_2_* ^2^A_1_ resonances. Thus, these states cannot lead to dissociation and are bound to relax through autodetachment. Furthermore, the lowest electronic excitation in benzonitrile is expected to be at around 3.5 eV (triplet state).^[^ ^42^, ^43^ ^]^ This imposes the lack of significant contributions from core‐excited configurations to the ion yield observed at about 3 eV. The CN^−^ contribution peaking at 3.0 eV must therefore be from formation of one or more of the π_3_* ^2^B_2_‐, π_4_* ^2^B_1_‐, and/or σ* ^2^A_1_‐negative ion states.

In general, for energetically opened channels, DEA may be a direct process through single‐electron occupation of a repulsive σ* state, as would be the case for the σ* ^2^A_1_ resonance. In the current calculations, however, the obtained lifetime of the σ* ^2^A_1_ state at fixed nuclei is about 0.4 fs, which makes such direct dissociation from this state unlikely. Nonadiabatic π*/σ* coupling may also lead to the dissociative decay of a π* state along a repulsive σ* coordinate if coupling of the respective vibrational modes is provided. Particularly for the π_4_* resonance, according to elementary selection rules, the π_4_*/σ* nonadiabatic coupling should be mediated by a normal coordinate belonging to the b1 symmetry component. Therefore, we assign the peak at ≈3.0 eV in the CN^−^ ion yield to the π_4_* ^2^B_1_ resonance, which then decays along the σ* C—CN coordinate through nonadiabatic π_4_*/σ* coupling. This is also in good agreement with the study by Heni and Illenberger^[^ ^17^ ^]^ on benzonitrile, where they found virtually no kinetic energy (KE) release in any of the observed dissociation channels. The low KE release suggests indirect dissociation processes with effective intramolecular vibrational energy redistribution, rather than direct dissociation along a repulsive state, leading the authors to propose the current supported π_4_*/σ* nonadiabatic coupling mechanism rather than direct dissociation from the σ* state.

The assignment of the high‐energy contributions in the CN^−^ ion yield to the respective negative ion states is more complicated. These are likely to include core‐excited anionic states, and CASPT2 calculations show a high density of electronic states for the benzonitrile anion in the energy range from 5 to 13 eV. However, (ππ_3_*)π_2_* and (ππ_3_*)σ* doublets, belonging to the overall A_2_ and A_1_ symmetry components, respectively, stand out in the 7–9 eV energy range and are likely to contribute to the structures observed in the DEA signals of benzonitrile. In this notation, the (ππ_3_*) term in the main configuration denotes a single excitation in the B_2_ component, and a single‐electron occupation in the π_2_* and σ* orbitals, respectively.

## 4 Conclusion

The present work reports DEA experiments on benzonitrile in the energy range 0–30 eV. The dominant fragment anion is CN^−^, which is formed through resonant contributions around 3.0 eV and over an extended range from about 7 to 10 eV in good agreement with Heni and Illenberger's earlier experiments.^[^ ^17^ ^]^ We ran the measurements up to 30 eV to check for possible DD channels, but no negative ion formation was observed. We present the most in‐depth theoretical study to date of the anionic states in the vicinity of the 3.0 eV experimental DEA structure using the SMCPP^[^ ^44^ ^]^, as well as virtual orbital scaling approach. In good agreement with the ET spectra of benzonitrile,^[^ ^16^ ^]^ four π* states, belonging to the A_2_, B_1_, and B_2_ symmetry components, were identified below about 5 eV as well as a σ* resonance with a VAE of 2.83 eV (SMCPP). Based on these calculations, the CN^−^ contribution can be assigned to a ^2^B_1_ resonance located at 3.19 eV in the SMCPP approach that dissociates along the σ* C—CN coordinate through adiabatic vibronic coupling with the σ* state. For the higher energy range, i.e., about 6 to 12 eV, the calculations do not offer a clear picture; however, complete active space plus second‐order perturbation (CASPT2) calculations imply strong contributions from core‐excited resonances with single‐electron occupation of the π_4_* and σ* identified with the SMCPP approach.

## Conflict of Interest

The authors declare no conflict of interest.

## Data Availability

The data that support the findings of this study are openly available in Zenodo at https://doi.org/10.5281/zenodo.17091717, reference number.^[^ ^45^ ^]^

## References

[1] P. Quadrelli , P. Caramella , Curr. Org. Chem. 2007, 11, 959.

[2] D. B. Bagal , B. M. Bhanage , Adv. Synth. Catal. 2015, 357, 883.

[3] S. Y. Byeon , J. Y. Lee , Dyes Pigm. 2016, 130, 183.

[4] B. A. McGuire , A. M. Burkhardt , S. Kalenskii , C. N. Shingledecker , A. J. Remijan , E. Herbst , M. C. McCarthy , Science 2018, 359, 202. 29326270 10.1126/science.aao4890

[5] J. K. Jørgensen , A. Belloche , R. T. Garrod , Annu. Rev. Astron. Astrophys. 2020, 58, 727.

[6] D. Bou Debes , M. Mendes , R. Rodrigues , J. Ameixa , L. M. Cornetta , F. Ferreira da Silva , S. Eden , Astron. Astrophys. 2025, 693, A304.

[7] G. Wenzel , I. R. Cooke , P. B. Changala , E. A. Bergin , S. Zhang , A. M. Burkhardt , A. N. Byrne , S. B. Charnley , M. A. Cordiner , M. Duffy , Z. T. P. Fried , H. Gupta , M. S. Holdren , A. Lipnicky , R. A. Loomis , H. T. Shay , C. N. Shingledecker , M. A. Siebert , D. A. Stewart , R. H. J. Willis , C. Xue , A. J. Remijan , A. E. Wendlandt , M. C. McCarthy , B. A. McGuire , Science 2024, 386, 810. 39446895 10.1126/science.adq6391

[8] G. Wenzel , T. H. Speak , P. B. Changala , R. H. J. Willis , A. M. Burkhardt , S. Zhang , E. A. Bergin , A. N. Byrne , S. B. Charnley , Z. T. P. Fried , H. Gupta , E. Herbst , M. S. Holdren , A. Lipnicky , R. A. Loomis , C. N. Shingledecker , C. Xue , A. J. Remijan , A. E. Wendlandt , M. C. McCarthy , I. R. Cooke , B. A. McGuire , Nat. Astron. 2024, 9, 262.

[9] G. Wenzel , S. Gong , C. Xue , P. B. Changala , M. S. Holdren , T. H. Speak , D. A. Stewart , Z. T. P. Fried , R. H. J. Willis , E. A. Bergin , A. M. Burkhardt , A. N. Byrne , S. B. Charnley , A. Lipnicky , R. A. Loomis , C. N. Shingledecker , I. R. Cooke , M. C. McCarthy , A. J. Remijan , A. E. Wendlandt , B. A. McGuire , Astrophys. J. Lett. 2025, 984, L36. 41103619 10.3847/2041-8213/adc911 PMC12523932

[10] G. Wenzel , M. Jiménez‐Redondo , M. Ončák , B. A. McGuire , S. Brünken , P. Caselli , P. Jusko , J. Phys. Chem. Lett. 2025, 16, 3938. 40211488 10.1021/acs.jpclett.5c00570 PMC12035856

[11] M. Agúndez , J. Cernicharo , M. Guélin , C. Kahane , E. Roueff , J. Kłos , F. J. Aoiz , F. Lique , N. Marcelino , J. R. Goicoechea , M. González García , C. A. Gottlieb , M. C. McCarthy , P. Thaddeus , Astron. Astrophys. 2010, 517, L2.

[12] B. N. Khare , C. Sagan , J. E. Zumberge , D. S. Sklarew , B. Nagy , Icarus 1981, 48, 290.

[13] D. B. Rap , J. G. M. Schrauwen , B. Redlich , S. Brünken , Phys. Chem. Chem. Phys. 2024, 26, 7296. 38353151 10.1039/d3cp05574d PMC10900304

[14] D. B. Rap , A. Simon , K. Steenbakkers , J. G. M. Schrauwen , B. Redlich , S. Brünken , Faraday Discuss 2023, 245, 221. 37404008 10.1039/d3fd00015j PMC10510038

[15] J. Kamer , D. Schleier , M. Donker , P. Hemberger , A. Bodi , J. Bouwman , Phys. Chem. Chem. Phys. 2023, 25, 29070. 37861750 10.1039/d3cp03977c

[16] P. D. Burrow , A. E. Howard , A. R. Johnston , K. D. Jordan , J. Phys. Chem. 1992, 96, 7570.

[17] M. Heni , E. Illenberger , Int. J. Mass Spectrom. Ion Process 1986, 73, 127.

[18] H. Abdoul‐Carime , G. Thiam , F. Rabilloud , ChemPhysChem 2024, 25, e303400287. 10.1002/cphc.202400287 38923142

[19] J. Langer , I. Dąbkowska , Y. Zhang , E. Illenberger , Phys. Chem. Chem. Phys. 2008, 10, 1523. 18327308 10.1039/b714320f

[20] G. W. Dillow , P. Kebarle , J. Am. Chem. Soc. 1989, 111, 5592.

[21] Albert Zlatkis , C. K. Lee , W. E. Wentworth , E. C. M. Chen , Anal. Chem. 1983, 55, 1596.

[22] W. E. Wentworth , L. W. Kao , R. S. Becker , J. Phys. Chem. 1975, 79, 1161.

[23] N. Kirnosov , L. Adamowicz , Chem. Phys. Lett. 2017, 676, 32.

[24] S. Gulania , T.‐C. Jagau , A. Sanov , A. I. Krylov , Phys. Chem. Chem. Phys. 2020, 22, 5002. 32077457 10.1039/c9cp06484b

[25] K. Andersson , P.‐Å. Malmqvist , B. O. Roos , J. Chem. Phys. 1992, 96, 1218.

[26] I. Fdez Galván , M. Vacher , A. Alavi , C. Angeli , F. Aquilante , J. Autschbach , J. J. Bao , S. I. Bokarev , N. A. Bogdanov , R. K. Carlson , L. F. Chibotaru , J. Creutzberg , N. Dattani , M. G. Delcey , S. S. Dong , A. Dreuw , L. Freitag , L. M. Frutos , L. Gagliardi , F. Gendron , A. Giussani , L. González , G. Grell , M. Guo , C. E. Hoyer , M. Johansson , S. Keller , S. Knecht , G. Kovačević , E. Källman , et al., J. Chem. Theory Comput. 2019, 15, 5925. 31509407 10.1021/acs.jctc.9b00532

[27] J. Pereira‐da‐Silva , R. Rodrigues , J. Ramos , C. Brígido , A. Botnari , M. Silvestre , J. Ameixa , M. Mendes , F. Zappa , S. J. Mullock , J. M. M. Araújo , M. T. do , N. Varella , L. M. Cornetta , F. F. da Silva , J. Am. Soc. Mass Spectrom. 2021, 32, 1459. 33998788 10.1021/jasms.1c00057

[28] A. Stamatovic , G. J. Schulz , Rev. Sci. Inst. 1970, 41, 423.

[29] D. Klar , M.‐W. Ruf , H. Hotop , Int. J. Mass Spectrom. 2001, 205, 93.

[30] M. H. F. Bettega , A. P. P. Natalense , M. A. P. Lima , L. G. Ferreira , Int. J. Quantum Chem. 1996, 60, 821.

[31] T. H. Dunning , J Chem Phys 1970, 53, 2823.

[32] R. F. da Costa , M. T. do N. Varella , M. H. F. Bettega , M. A. P. Lima , Eur. Phys. J. D 2015, 69, 159.

[33] C. W. Bauschlicher , J. Chem. Phys. 1980, 72, 880.

[34] F. Kossoski , M. H. F. Bettega , J. Chem. Phys. 2013, 138, 234311. 23802964 10.1063/1.4811218

[35] M. J. Frisch , G. W. Trucks , H. B. Schlegel , G. E. Scuseria , M. A. Robb , J. R. Cheeseman , G. Scalmani , V. Barone , G. A. Petersson , H. Nakatsuji , X. Li , M. Caricato , A. Marenich , J. Bloino , B. G. Janesko , R. Gomperts , B. Mennucci , H. P. Hratchian , J. V. Ortiz , A. F. Izmaylov , J. L. Sonnenberg , D. Williams‐Young , F. Ding , F. Lipparini , F. Egidi , J. Goings , B. Peng , A. Petrone , T. Henderson , D. Ranasinghe , et al., Gaussian, Inc. Wallingford CT 2016.

[36] L. A. Curtiss , P. C. Redfern , K. Raghavachari , J. Chem. Phys. 2007, 127, 124105. 17902891 10.1063/1.2770701

[37] L. A. Curtiss , P. C. Redfern , K. Raghavachari , J. Chem. Phys. 2007, 126, 084108. 17343441 10.1063/1.2436888

[38] G. Li Manni , I. Fdez. Galván , A. Alavi , F. Aleotti , F. Aquilante , J. Autschbach , D. Avagliano , A. Baiardi , J. J. Bao , S. Battaglia , L. Birnoschi , A. Blanco‐González , S. I. Bokarev , R. Broer , R. Cacciari , P. B. Calio , R. K. Carlson , R. Carvalho Couto , L. Cerdán , L. F. Chibotaru , N. F. Chilton , J. R. Church , I. Conti , S. Coriani , J. Cuéllar‐Zuquin , R. E. Daoud , N. Dattani , P. Decleva , C. de Graaf , et al., J. Chem. Theory Comput. 2023, 19, 6933.37216210

[39] J. Zhao , K. Zhang , X. Cheng , X. Yang , J. Mol. Struct. Theochem 2008, 863, 133.

[40] N. Kosar , K. Ayub , M. A. Gilani , T. Mahmood , J Mol Model 2019, 25, 47. 30690660 10.1007/s00894-019-3930-x

[41] S. E. Bradforth , E. H. Kim , D. W. Arnold , D. M. Neumark , J. Chem. Phys. 1993, 98, 800.

[42] B. N. Rajasekhar , V. Dharmarpu , A. K. Das , A. Shastri , A. Veeraiah , S. Krishnakumar , J. Quant. Spectrosc. Radiat. Transf. 2022, 283, 108159.

[43] R. C. Hirt , J. P. Howe , J. Chem. Phys. 1948, 16, 480.

[44] G. B. Bachelet , D. R. Hamann , M. Schlüter , Phys. Rev. B 1982, 26, 4199. 10.1103/physrevb.37.4798 9945148

[45] F. Ferreira da Silva , Revisiting CN− Formation Mechanisms in Electron Collisions with Benzonitrile [Data set]. Zenodo 2025, 10.5281/zenodo.17091717. PMC12810441 41016733

